# Rutin Supplementation Reduces Oxidative Stress, Inflammation and Apoptosis of Mammary Gland in Sheep During the Transition Period

**DOI:** 10.3389/fvets.2022.907299

**Published:** 2022-05-27

**Authors:** Hongyan Ding, Yu Li, Chang Zhao, Yue Yang, Chengkun Xiong, Daoliang Zhang, Shibin Feng, Jinjie Wu, Xichun Wang

**Affiliations:** College of Animal Science and Technology, Anhui Agricultural University, Hefei, China

**Keywords:** rutin, transition sheep, oxidative stress, inflammation, apoptosis

## Abstract

Rutin, a common dietary flavonoid, exhibits remarkable pharmacological activities such as antioxidant and anti-inflammatory functions. Metabolic stress in mammals during the transition period affects mammary gland health. The aim of this experiment was to evaluate the protective effect of rutin supplementing against metabolic stress in the mammary glands of sheep during the transition period, particularly after parturition. Transition *Hu* sheep (2–3 years old with 62.90 ± 2.80 kg) were randomly divided into three groups, the control group was fed a diet without rutin, while rutin (50 and 100 mg/kg body weight/day) was administered to the two treatment groups (−28 day to +28 day relative to parturition). Serum and blood samples were collected from jugular vein on days −14, −7, +1, +2, +7, +14, +21, +28 relative to parturition. Mammary tissue biopsy samples of four sheep from the treatment group were harvested on day +28 postpartum. Compared to that in the control group, rutin supplementation resulted in lower β-hydroxybutyrate (BHBA) while increasing the concentrations of non-esterified fatty acids (NEFA) and globulin after lactation. Furthermore, rutin treatment led to lower hydrogen peroxide (H_2_O_2_) and malonaldehyde (MDA) levels, resulting in increased catalase (CAT), glutathione peroxidase (GSH-Px), superoxide dismutase (SOD) and total antioxidant potential (T-AOC). Compared to that in the control group, rutin inhibits the mRNA expression of inflammatory markers such as tumor necrosis factor-α (*TNF-*α). In addition, rutin markedly downregulated the ratio of phosphorylated NF-κB p65 (p-p65) to total NF-κB p65 (p65). Meanwhile, rutin supplementation resulted in high mRNA abundance of the nuclear factor erythroid 2-like 2 (*NFE2L2*, formerly *NRF2*) and its target gene, heme oxygenase-1 (*HO-1*), which plays critical roles in maintaining the redox balance of the mammary gland. Furthermore, rutin treatment lowered the levels of various downstream apoptotic markers, including Bax, caspase3 and caspase9, while upregulating anti-apoptotic Bcl-2 protein. These data indicate the positive effect of rutin against inflammation, oxidative stress status, and anti-apoptotic activity in the mammary gland. The mechanism underlying these responses merits further study.

## Introduction

The transition period is defined as the 3 weeks before calving and the 3 weeks after calving and is one of the most critical periods in the life of sheep and many animals (1, 2). During the transition period, maternal animals undergo profound physiological alterations, which can drastically modify their metabolism (3, 4). However, when mammals fail to adapt physiologically to productive lactation, this may trigger metabolic stress, which is characterized by the combined effects of uncontrolled adipose tissue lipolysis, oxidative stress, and chronic inflammation (4, 5). Livestock undergo substantial metabolic stress that results in a range of postpartum health problems, which could significantly reduce animal welfare and increase economic losses (6).

Metabolic stress in ruminants is caused by a cluster of risk factors in the transition period, which can lead an increased incidence of certain health disorders such as mastitis, displaced abomasum, and ketosis (5). The physiological outcomes of metabolic stress in periparturient sheep are analogous to those of metabolic syndromes in humans (7, 8). Early lactating ewes experience aberrant nutrient metabolism when they cannot meet the nutrient requirements for milk synthesis and secretion through dietary intake. This is indicated by biomarkers of fat mobilization such as non-esterified fatty acids (NEFA) (4). Although lipid mobilization is a normal physiological response that helps ruminants adapt to a negative energy balance (NEB), excessive fat mobilization is a problem. The overproduction of ketones, such as β-hydroxybutyric acid (BHBA), can cause ketosis during times of high serum NEFA levels, which cannot be processed by hepatocytes (4, 8–11). Additionally, the increase in NEFA and BHBA has been linked to metabolic and infectious diseases during the transition period (12). Furthermore, aberrant nutrient metabolism, chronic inflammation, and oxidative stress can intensify other factors that can cause metabolic stress (5). In both humans and ruminants, increased serum NEFA levels can lead to increased reactive oxygen species (ROS) levels, which can exacerbate oxidative stress (13). Enhanced oxidative stress can contribute to additional lipolysis and dysregulated inflammatory responses (14). Measures of certain biomarkers are excellent predictors of the risk of diseases during the transition period (12–14).

It is well known that complex changes in the mammary gland during the last stage of pregnancy and the formation of colostrum begin a few weeks before calving (15–17). These changes imply increased production of ROS and cytokines in the breast and a higher risk of intramammary infection (13). Nutrition has a major impact on the health of udders (18).

Recently, the application of natural agents, such as flavonoids, as potential means of improving animal health has become more widespread (19). Rutin is a flavonoid found in many plants and has various beneficial health effects, including antioxidative, anti-inflammatory, antimicrobial, antitumor, and chelating properties, amongst others. In a study on aged rats, rutin supplementation reduced systemic inflammation and oxidative stress (20). Additionally, rutin inhibited the lipoteichoic acid-induced increase in ROS levels, levels of the pro-inflammatory enzyme cyclooxygenase-2, and phosphorylation of mitogen-activated protein kinases in H9c2 cells (21). Rutin is one of the most consumed flavonoids in human diet and has been used for clinical treatment of lymphedema and chylothorax (22, 23).

Owing their health-promoting properties, high safety margins, and low cost, flavonoids are used as feed supplements for ruminants (19). In lactating dairy cows, long-term dietary supplementation with rutin increases milk yield and improves metabolism and digestibility while elevating serum glucose, BHBA, and albumin levels (24, 25). These results indicate a possible metabolic effect of rutin on energy metabolism in ruminants. Quercetin is a wellknown, potent antioxidant. Quercetin is beneficial in periparturient, high-yielding dairy cows, as it ameliorates the negative effects of free radical formation (26). Rutin, the rhamnoglucoside of quercetin, which is likely a rumen-protecting source of quercetin, may be a better antioxidant than quercetin in ruminants when used orally (27).

However, little information is available regarding the effects of rutin on metabolic stress and mammary gland status during the transition period in sheep. Therefore, the present study aimed to identify whether rutin affects metabolic stress and mammary gland status in sheep during the transition period. We hypothesized that oral rutin supplementation would have beneficial effects on nutrient metabolism, inflammation, and oxidative stress in sheep during the transition period.

## Materials and Methods

### Experimental Design and Treatments

This study was conducted at a *Hu* sheep breeding and raising station in Maanshan, Anhui Province, China. Twenty-four estrus synchronized, clinically healthy 2 to 3-year-old *Hu* ewes, with an average bodyweight (BW) of 62.90 ± 2.80 kg (mean ± SE) were used in a randomized, complete, balanced block design experiment and divided into three groups, with eight *Hu* sheep per treatment group. Each group was divided into two cages during the experiment, with four sheep per cage. The ewes in the control group were offered a basal control diet (control), and the ewes in the rutin treatment groups were offered a basal diet plus rutin (50 or 100 mg/kg of body weight; rutin, Target Molecule Corp., Wellesly Hills, MA). The trial lasted for the period from day −28 to +28 relative to parturition. The treatment diets were administered twice a day at 06:00 and 15:00. The ingredients and chemical compositions of the diets are listed in Table 1. The sheep had *ad libitum* access to hay and water and were housed in a free-stall barn cooled by fans.

**Table 1:** The ingredients and chemical composition of the diets (g/100 g of concentrate).

**Chemical composition**	**Concentrate**	**Hay**
Crude protein	14	6.86
Calcium (mg/kg)	2.64 × 10^5^	1.74 × 10^5^
Phosphorus (mg/kg)	9.98 × 10^3^	2.23 × 10^3^
Crude fat	1.4	0.6
Dry matter	88.2	88.6
Acid detergent fiber	3.0	43.9
Neutral detergent fiber	67.0	60.9

*The premix provided the following per kg of diets: Fe 24 mg, Cu 7 mg, Mn 39 mg, Zn 41 mg, I 0.5 mg, Se 0.2 mg, Co 0.1 mg, VD 110 IU, VA 938 IU, VE 18 IU*.

### Blood Parameters Determination

Blood samples were collected from the jugular vein using evacuated tubes, containing lithium heparin, before feeding on days −14, −7, +1, +2, +7, +14, +21, and +28 relative to parturition. Samples were immediately stored on ice throughout the collection and processing stages. Plasma was harvested *via* centrifugation at 1,500 × *g* and 4°C for 15 min. Plasma aliquots were frozen at −80 °C for further analysis. BHBA (146521007), calcium (142220009), albumin (148321002), total protein (TP, 140821002), triglyceride (TG, 141721006), total cholesterol (TC, 141721006), and fructosamine (FUN, 145421004) concentrations were analyzed using a Mindray automatic Chemistry Analyzer (Mindray, Shenzhen, China) and commercial kits. Globulin content was calculated by subtracting the albumin concentration from the total protein concentration. Additional aliquots of plasma were pending analysis within 1 month of collection for oxidative stress indicators. Commercial assays were used to quantify oxidative stress indices according to the manufacturer's instructions. The serum total antioxidant potential concentration (T-AOC) was determined using a sheep T-AOC ELISA Kit (HY72220-A; Shanghai Enzyme-linked Biotechnology Co., Ltd., Shanghai, China). The levels of superoxide dismutase (SOD, HY72228-A), glutathione peroxidase (GSH-Px, H72232-A), catalase (CAT, HY72138-A), malonaldehyde (MDA, HY72142-A), and hydrogen peroxide (H_2_O_2_, HY72224-A) in the serum were determined using an ELISA kit from Shanghai Enzyme-linked Biotechnology (Shanghai, China) according to the manufacturer's protocols. Whole-blood samples were collected in EDTA-evacuated tubes and refrigerated at 4°C immediately upon returning from the farm until blood leukocyte analysis could be performed. Total leukocytes were quantified on collection days using an automated routine blood test (Mindray, Shenzhen, China).

### Mammary Gland Samples Collection

Mammary gland tissues were harvested on day +28 from a subset of two sheep in each cage *via* percutaneous biopsy. The procedure was conducted under mild general anesthesia with xylazine and local anesthesia with lidocaine HCl. A 3 cm incision was made through the skin and subcutaneous tissue and then separated from the mammary capsule at the incision site. Approximately 200 mg of tissue was removed using a biopsy needle (Bard Magnum, 12 gauge × 16 cm; C. R. Bard Inc., Murray Hill, NJ), immediately snap-frozen in liquid nitrogen, and stored at −80°C until further analysis.

### RNA Isolation, CDNA Synthesis and Quantitative RT-PCR

Total RNA was isolated from 50–100 mg of mammary gland tissue using TRIzol (Life Technologies, Grand Island, NY) according to the manufacturer's protocols. The RNA samples were reverse-transcribed using the PrimeScript™RT reagent Kit with gDNA Eraser (Takara Bio, Dalian, China). The quality of RNA samples was measured using an Agilent 2100 Bioanalyzer (Agilent Technologies, Santa Clara, CA). All samples had an RNA integrity number factor >7.3. β-actin was used as the internal control gene to normalize the mRNA expression. Primers were designed and synthesized by Sangon Biotechnology Co. Ltd. (Shanghai, China) based on the sequences reported in GenBank. The primer sequences used in this study are listed in Table 2. qRT-PCR was carried out using Novostart SYBR qPCR SuperMix Plus (Novoprotein, Fremont, CA) in an ABI 7500 fast Real-Time PCR machine (Applied Biosystems, Waltham, MA) following the manufacturer's instructions. cDNA from 1 μg total RNA was used as the template; the following cycling conditions were used: 95°C for 1 min for initial denaturation and enzyme activation, 40 cycles of 95°C for 20 s and 60°C for 1 min for amplification. The 2^−Δ*ΔCT*^ method was used to calculate the relative gene expression with data from samples collected at enrolment (baseline sample) as calibrator samples.

**Table 2:** Primer sequences of the genes.

**Genes**		**Primer sequences (5^**′**^-3^**′**^)**	**Length (bp)**
IL-1β	Forward	GGGTATCAGGGACAAGAATC	181
	Reverse	CCAGTTAGGGTACAGGACAG	
IL-6	Forward	TTCCAATCTGGGTTCAATCA	104
	Reverse	TTTCCCTCAAACTCGTTCTG	
TNF-a	Forward	AAAGACAGCATGAGCACCAA	110
	Reverse	CTGAGGCACCAGCAACTTCT	
AMPKα	Forward	GAAGATCGGTCACTACATCCT	75
	Reverse	TCATGTTTGCCAACCTTCAC	
NFE2L2	Forward	CCACATTCCCAAAGCAGATG	118
	Reverse	GGGAACAGGTGATTGAAACG	
HMOX1	Forward	CGAGAAGGTTTTAAGCTGGTG	80
	Reverse	TCCTTGTTGCGTTCGATCT	
NQO1	Forward	TTCAATCCCGTCATCTCCAG	77
	Reverse	GTCTCGGCAGGATACTGAAA	
β-actin	Forward	TCCTTCCTGGGCATGGAATC	91
	Reverse	CGTAAAGGTCCTTGCGGATG	

### Western Blot Analysis

Proteins were extracted from mammary gland tissue (50–100 mg) using radioimmuno precipitation assay (RIPA) lysis buffer (C500007; Sangon Biotech, Shanghai, China) according to Li et al. (28). Protein concentrations were measured using a BCA Protein Assay Kit (E-BC-K318; Elabscience, Houston, TX). Total protein (40 μg per lane) was loaded on a 12% SDS-PAGE gel and run at 80 V for 30 min and 120 V for 1.5 h. The resolved proteins were electrotransferred onto 0.45 μm polyvinylidene fluoride (PVDF) membranes. PVDF membranes were blocked in Tris-buffered saline (TBS), containing 0.1% Tween-20 (TBST) and 5% skim milk powder (A600669-0250; Sangon Biotech, Shanghai, China), for 2 h at room temperature. The blocked membranes were then incubated overnight at 4°C in TBST containing primary antibodies against adenosine monophosphate-activated protein kinase (AMPK, 5831; CST, Danvers, MA), p-AMPKα (2535; CST, Danvers, MA), nuclear factor erythroid 2-related factor 2 (NFE2L2, 16396-1-ap; Proteintech group, Chicago, IL), inhibitor of kappa B α (IκBα, GB13212-1; Servicebio, Wuhan, China), p-IκBα (2859; CST, Danvers, MA), p65 (380172; Zen Bioscience, Chengdu, China), p-p65 (AF2006; Affinity Bioscience, Changzhou, China), B cell leukemia/lymphoma-2 (Bcl-2, BF9103; Affinity Bioscience, Changzhou, China), Bcl-2-associated X protein (Bax, R22708; Zen Bioscience, Chengdu, China), cysteine-containing aspartate-specific proteases-3 (caspase3, 383315; Zen Bioscience, Chengdu, China), and cysteine-containing aspartate-specific proteases-9 (caspase9, AF6348; Univ Biotechnology, Shanghai, China). The membranes were then washed six times with TBST and incubated with horseradish peroxidase-conjugated anti-mouse or anti-rabbit secondary antibodies (BA1050 and BA1054; Boster, Wuhan, China) for 1 h at room temperature. The membranes were washed with TBST and then incubated with an enhanced chemiluminescence reagent (32109; Thermo Scientific, Shanghai, China). β-actin (200068-8F10; Zen Bioscience, Chengdu, China) was used as an internal control. Images were captured using a ChemiDOC XRS imaging system (Bio-Rad, Hercules, CA). The intensities of the bands were quantified using the Image Lab Software (version 5.2.1, Bio-Rad) and normalized to the intensity of β-actin (29).

### Statistical Analysis

The data were analyzed using the MIXED procedure of SAS v.9.4 (SAS Institute Inc., Cary, NC) according to the following model:

Y_ij_ = μ + b_i_ + M_j_ + e_ij_,

where Y_ij_ = dependent, continuous variable, μ = overall mean, b_i_ = random effect of block, M_j_ = fixed effect of treatment (j = control vs. rutin), and e_ij_ = residual error. Normality of the residuals was checked with normal probability and box plots and homogeneity of variances with plots of residuals vs. predicted values. The data for mRNA and protein expression were analyzed using the One-way ANOVA procedure of SPSS 19.0 (SPSS 19.0, SPSS Inc., Chicago, IL, USA). Values were expressed as mean ± SE. Significance was determined at *P* < 0.05. Graphs were generated by GraphPad Prism 8 (La Jolla, CA, USA).

## Results

### Effects of Rutin Supplementation to Hu Sheep From −14 Through +28 d Around Parturition on Nutrient Metabolism Biomarkers

As shown in Table 3, nutrient metabolism biomarkers varied over the sampling period. The sheep with 100 mg/kg rutin supplementation tended to have lower concentration of BHBA (*P* = 0.02) and higher concentration of NEFA (*P* = 0.05) in the plasma. No differences (*P* > 0.05) between groups were observed for Calcium, TG, TC, and FUN. A time effect (*P* < 0.05) was observed for the concentration of BHBA, NEFA, TG, TC, and FUN.

**Table 3:** Effects of rutin supplementation to Hu sheep from −14 through 28 d around parturition on biomarkers of nutrient metabolism.

	**Diet**				***P*** **-value**		
**Items**	**Control**	**50 mg/kg rutin**	**100 mg/kg rutin**	**SE**	**Treatmen^a^**	**Time**	**Treatment × Time**
BHBA (mmol/L)	0.78	0.66	0.58	0.06	0.06	<0.01	0.40
NEFA (μmol/L)	1,292.51	1,228.65	1,372.91	51.73	0.16	<0.01	<0.01
Calcium (mmol/L)	2.22	2.23	2.18	0.05	0.75	0.27	0.82
TG (mmol/L)	0.27	0.31	0.29	0.01	0.11	<0.01	0.32
TC (mmol/L)	1.78	1.93	1.93	0.13	0.60	<0.01	0.93
FUN (μmol/L)	179.58	182.25	188.99	6.41	0.56	<0.01	0.61

a *Rutin effect*.

### Effects of Rutin Supplementation to Hu Sheep From −14 Through +28 d Around Parturition on Serum Indicators Inflammation

As shown in Table 4, sheep with 50 mg/kg rutin supplementation tended to have higher content of Globulin (*P* = 0.05) in the plasma. No differences (*P* > 0.05) between groups were observed for Albumin and Leukocyte. A time effect (*P* < 0.05) was observed for the content of Albumin and Globulin.

**Table 4:** Effects of rutin supplementation to Hu sheep from−14 through 28 d around parturition on biomarkers of inflammation.

	**Diet**				***P*** **-value**		
**Items**	**Control**	**50 mg/kg rutin**	**100 mg/kg rutin**	**SE**	**Treatment**	**Time**	**Treatment × Time**
Albumin (g/L)	20.59	21.13	21.13	0.57	0.74	<0.01	0.27
Globulin (g/L)	45.30	49.60	43.99	1.50	0.02	<0.01	0.57
Leukocyte (10^9^/L)	9.96	9.88	13.73	2.63	0.47	0.27	0.53

### Effects of Rutin Supplementation to Hu Sheep From −14 Through +28 d Around Parturition on Serum Oxidative Stress Indicators

As shown in Table 5, sheep with rutin supplementation tended to have lower concentration of hydrogen peroxide (H_2_O_2_) (*P* < 0.01) and activity of MDA (*P* < 0.01). While sheep with rutin supplementation tended to have higher activity of CAT (*P* < 0.01), GSH-Px (*P* < 0.01), SOD (*P* < 0.01), and T-AOC (*P* < 0.01). A time effect (*P* < 0.01) was observed for the concentration and activity of H_2_O_2_, CAT, MDA, GSH-Px, SOD, and T-AOC.

**Table 5:** Effects of rutin supplementation to Hu sheep from−14 through 28 d around parturition on biomarkers of oxidative stress.

	**Diet**				***P*** **-value**		
**Items**	**Control**	**50 mg/kg rutin**	**100 mg/kg rutin**	**SE**	**Treatment**	**Time**	**Treatment × Time**
H_2_O_2_ (μmol/L)	68.26	62.22	54.83	1.47	<0.01	<0.01	<0.01
CAT (U/mL)	45.97	56.03	67.55	1.11	<0.01	<0.01	<0.01
MDA (μmol/L)	6.69	6.00	4.83	0.11	<0.01	<0.01	0.01
GSH-Px (U/mL)	281.11	363.15	418.10	8.57	<0.01	<0.01	<0.01
SOD (U/mL)	120.66	148.25	172.80	2.66	<0.01	<0.01	0.23
T-AOC (U/mL)	13.83	16.39	16.99	0.23	<0.01	<0.01	<0.01

### The Protein and MRNA Expression in Mammary Gland After Rutin Supplementation

**Figure 1:**
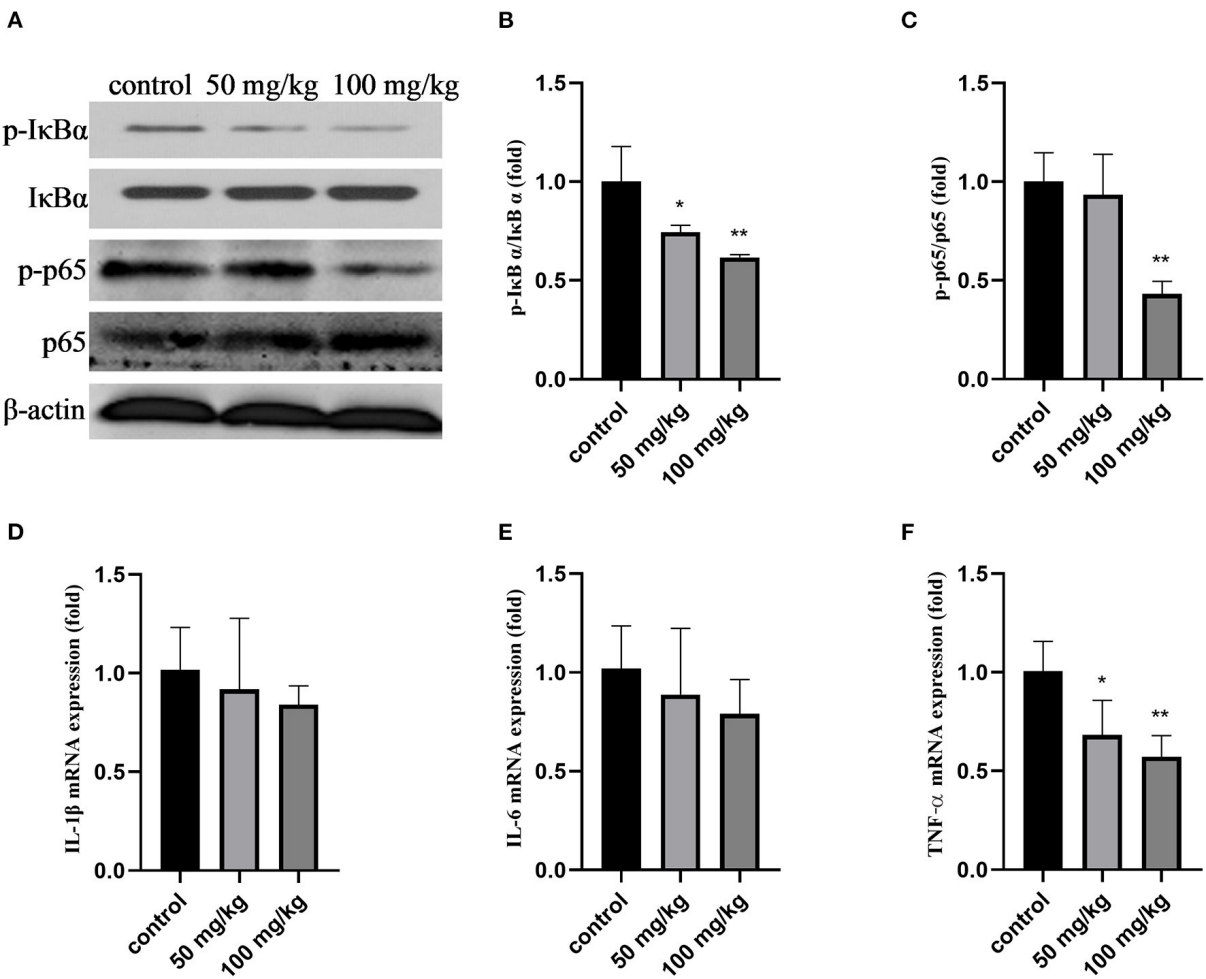
Effects of rutin supplementation on inflammation of mammary gland in sheep during the transition. **(A)** Western blot analysis of p-IκBα, IκBα, p-p65, and p65. **(B)** Ratio of p-IκBα/IκBα. **(C)** Ratio of p-p65/p65. **(D–F)** Relative mRNA abundance of *IL-1*β, *IL-6* and *TNF-*α. Data are presented as means ± SE. *P < 0.05, **P < 0.01.

As shown in Figures 1A–C,F, compared with that in the control group, the expression of p-IκBα, p-p65, the ratio of p-IκBα/IκBα, p-p65/p65, and the mRNA expression of tumor necrosis factor-α (TNF-α) decreased after rutin treatment (*P* < 0.05). Rutin supplementation also reduced the mRNA expression of interleukin-1β (*IL-1*β) and interleukin-6 (*IL-6*); however, the difference was not significant (Figures 1D,E). These results indicated that rutin supplementation suppressed the NF-κB signaling pathway.

**Figure 2:**
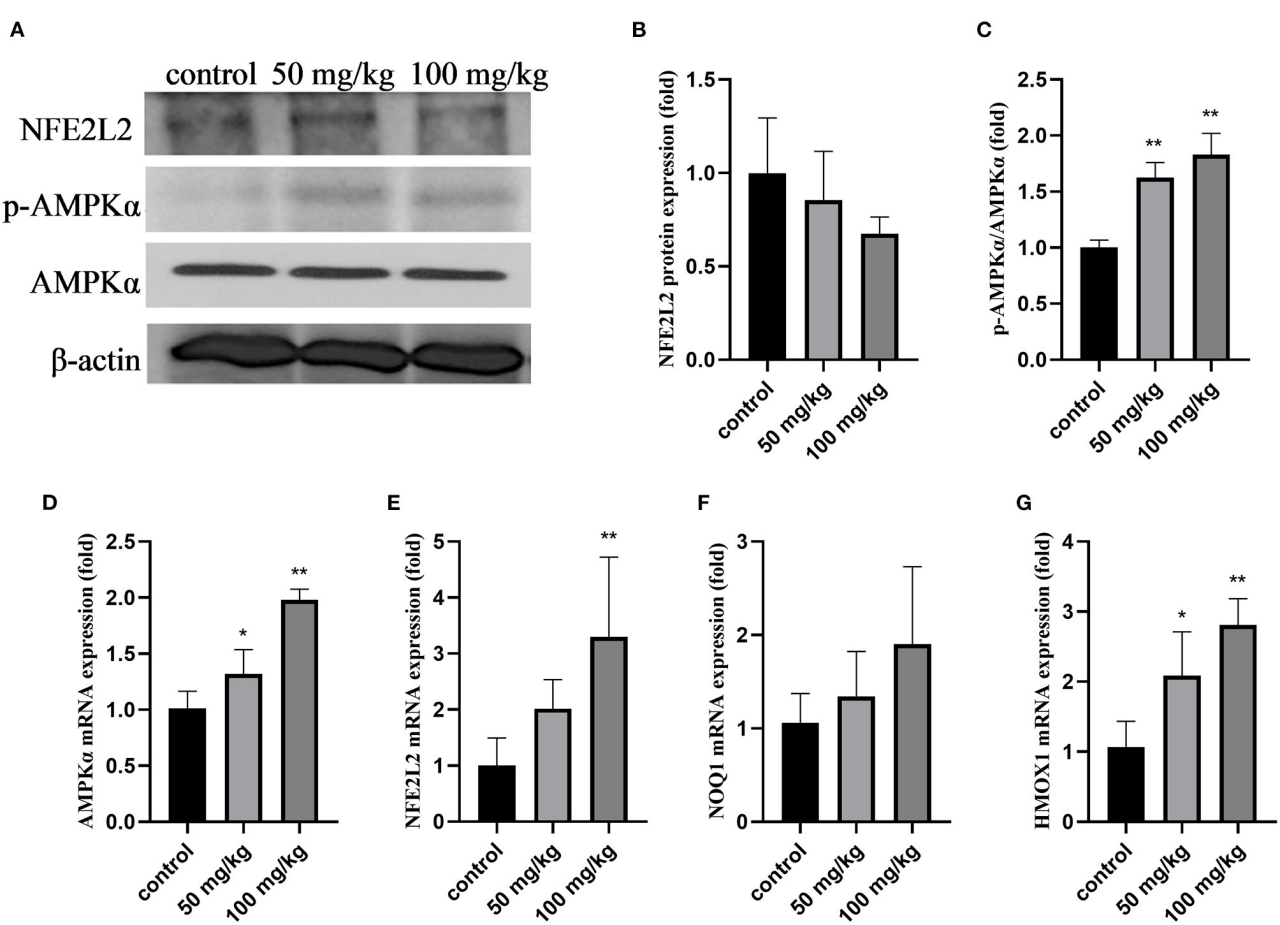
Effects of rutin supplementation on oxidative stress in the mammary gland in sheep during the transition. **(A)** Western blot analysis of NFE2L2, p-AMPKα and AMPKα. **(B)** Relative protein abundance of NFE2L2. **(C)** Relative protein abundance of p-AMPKα/AMPKα. **(D–G)** Relative mRNA abundance of *AMPK*α, *NFE2L2, NOQ1*, and *HMOX1*. Data are presented as means ± SE. *P < 0.05, **P < 0.01.

As shown in Figure 2, compared to that in the control group, rutin supplementation reduced the total protein expression of NFE2L2, but the difference was not significant (Figures 2A,B; *P* > 0.05). The expression of p-AMPKα and the ratio of p-AMPKα/AMPKα were significantly increased by rutin supplementation (Figures 2A,C; *P* < 0.01). The mRNA expression levels of *AMPK*α, *NFE2L2*, and *heme oxygenase 1* (*HMOX1*) were significantly increased by rutin supplementation (Figures 2D,E,G; *P* < 0.05 or *P* < 0.01). The mRNA expression of quinone oxido-ereductase 1 (*NOQ1*) was higher after rutin supplementation, but the difference was not statistically significant (*P* > 0.05). These results indicate that rutin supplementation activated the AMPK/NFE2L2 pathway.

**Figure 3:**
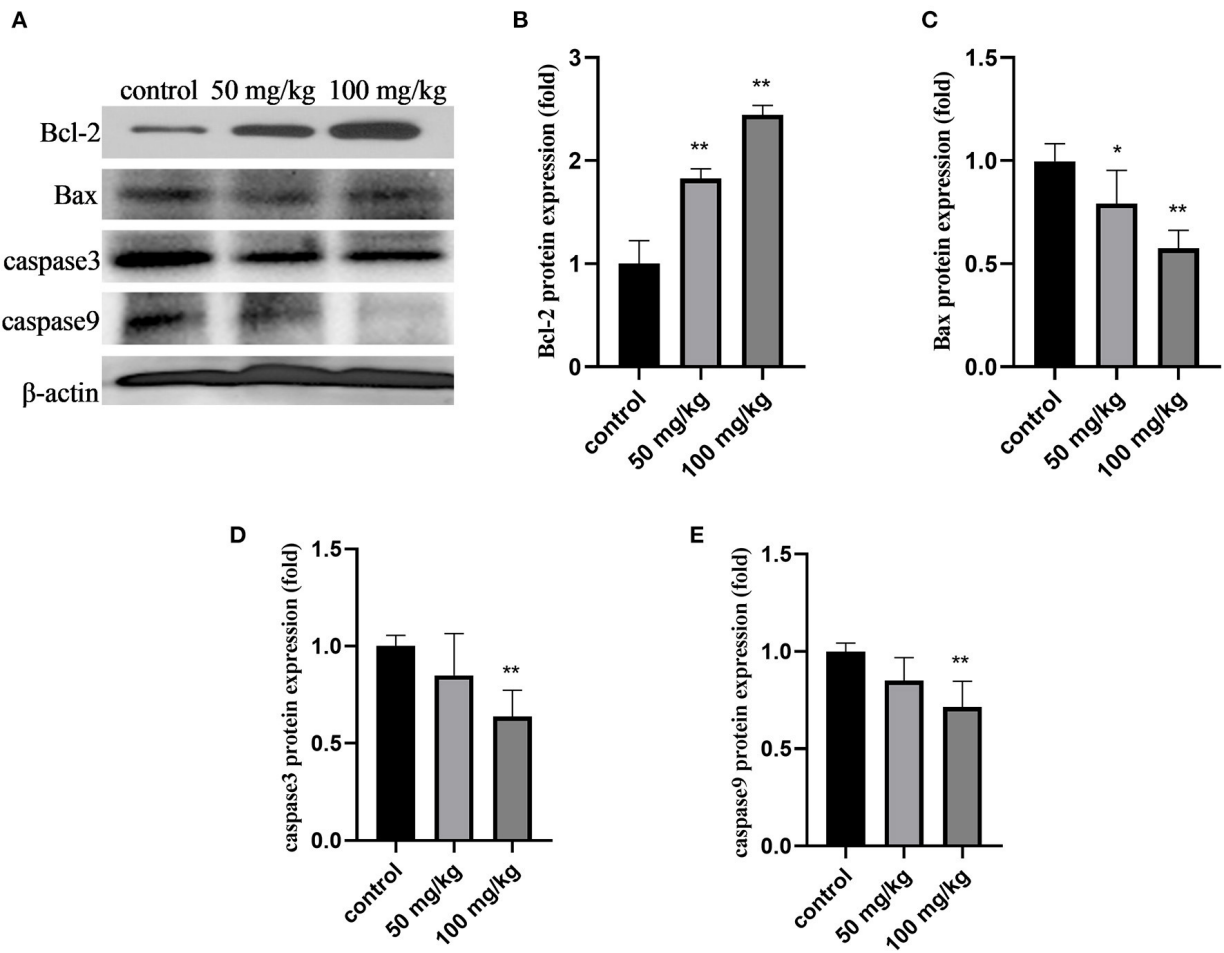
Effects of rutin supplementation on apoptosis of mammary gland in sheep during the transition. **(A)** Western blot analysis of Bcl-2, Bax, caspase3 and caspase9. **(B–E)** Relative protein abundance of Bcl-2, Bax, caspase3 and caspase9. Data are presented as means ± SE. *P < 0.05, **P < 0.01.

As shown in Figure 3, compared to that in the control group, 50 mg/kg and 100 mg/kg rutin supplementation increased the protein expression of Bcl-2 (Figures 2A,B; *P* < 0.01). Rutin (50 mg/kg) supplementation significantly lowered Bax protein expression (Figure 2C; *P* < 0.05). Furthermore, rutin (100 mg/kg) supplementation decreased the protein expression of Bax, caspase3, and caspase9 (Figures 3A,C,D,E; *P* < 0.01). These results indicate that rutin supplementation downregulates the caspase pathway.

## Discussion

Rutin, a flavonol widely present in various fruits and vegetables, has significant antioxidant properties (29). In *in vivo* studies, rutin showed a positive impact on energy metabolism and milk yield (24). *In vitro* studies with bovine mammary cells have revealed that the supply of several flavonols protects against oxidative damage by increasing NFE2L2 expression (30, 31). However, whether rutin elicits similar effects *in vivo* is unknown. In the present study, we investigated the effects of rutin on metabolic stress and mammary gland in sheep during the periparturient period.

### Serum Biomarkers

After the sheep were administered rutin, the serum levels of the biomarkers of nutrient metabolism, inflammation, and oxidative stress changed during the early lactation period, particularly in the first 2 weeks after calving. Serum NEFA and BHBA levels are helpful for energy status monitoring in pregnat ewes (32, 33). Elevated serum NEFA levels indicate increased fat mobilization (12). Fat mobilization is a normal physiological response that helps ruminants to adapt to NEB during the transition period. However, excessive fat mobilization can be problematic. NEFA > 400 μmol/L is characterized by high lipomobilization and values of BHBA are 0.8–1.2 mmol/L are indicative of NEB (34, 35). The serum concentrations of NEFA and BHBA are highly associated with hepatic lipidosis and other metabolic disorders (36). In the present study, the serum concentrations of NEFA were reduced before lactation and elevated after lactation by rutin supplementation at 100 mg/kg of body weight in sheep. However, rutin supplementation tended to reduce the plasma BHBA levels, which is in line with a previous study on dairy cows (6). These differences can be attributed to the sheep with rutin supplementation having an improved energy balance during lactation, and there might be enough carbohydrates available to fully oxidize NEFA, as opposed to shifting to ketone synthesis *via* incomplete oxidation. In addition, plasma TG and TC levels significantly decreased among the three groups from day −14 to day +14. These results are in accordance with those of a previous study on Makouei breed sheep (7). The serum FUN levels increased slightly; that is, rutin treatment increased fat mobilization but did not increase serum TG and ketone levels. The increase in NEFA levels may be due to the increased demand for glucose caused by increased lactation; this indicates that rutin exerts beneficial effects on energy metabolism, which is consistent with the findings of Stoldt et al. (6). The mechanisms underlying these effects need to be verified through future experiments.

In studies on dairy cows and goats, altered nutrient metabolism is related to many metabolic and inflammatory conditions, while the increase in the levels of certain biomarkers, such as NEFA and BHBA, has been linked to early lactation disease and altered immune competence (4, 5). In this study, the levels of the serum biomarkers of inflammation did not fluctuate significantly and were relatively stable with rutin supplementation after calving. This may be advantageous to ruminants during the transition period.

Oxidative stress occurs when there is an imbalance between ROS and antioxidant defenses and is a common disorder during periods of increased metabolic demands, such as the transition period (6, 37). To maintain antioxidant defenses against increasing ROS levels, it is common to add antioxidant supplements to periparturient diets (5, 38). Sgorlon S et al. found that dietary antioxidants (tomato pomace (1.3% lycopene) or grape skin (87% polyphenols)) had no effect on blood MDA, GSx and GPx (39). However, the findings indicate that antioxidants (vitamin E and Se) supplementation reduced the oxidative stress induced by heat stress (40). From the aboved findings, As a member of antioxidants, rutin has been shown to influence enzymes involved in scavenging ROS in monogastric animals *in vivo* and *in vitro* (41, 42). Thus, further researches are needed to investigate the real effect of dietray rutin (antioxidants) on sheep. In the present study, we measured the activities of enzymes involved in oxidative stress in the serum. SOD, CAT, and GSH-Px, the major antioxidant enzymes in the body, inhibit ROS-mediated toxicity by catalyzing the conversion of superoxide anions to hydrogen peroxide and oxygen, or by directly reacting with ROS (43). The levels of these enzymes involved in free-radical defense increased after rutin supplementation. Serum MDA levels were reduced and T-AOC levels were significantly increased after rutin supplementation. H_2_O_2_ is a major component of ROS (44). Therefore, we measured H_2_O_2_ content in the serum and found that rutin supplementation reduced the content of serum H_2_O_2_. These results indicate that oral rutin supplementation alleviates oxidative stress in sheep during the transition period. This is consistent with the results of previous studies on quercetin (45, 46), a monomer of rutin.

### Mammary Gland Status

Dramatic changes in growth, reorganization, and function of the mammary gland are prerequisites for milk production and are critical for nurturing offspring (47). The transition period is the most critical period during lactation in dairy cows. The mammary gland, the organ for milk production, undergoes faces physiological challenges associated with parturition and initiation of lactation (48). In this study, we examined the protective effects of rutin against inflammation, oxidative stress, and apoptosis in the mammary glands of sheep during the transition period. Our data showed that rutin supplementation improved the mammary gland status.

Dairy cows may undergo an overt systemic inflammatory response around the time of calving, even if there are no signs of microbial infections or other pathologies (49). In this study, we investigated the effects of rutin supplementation on the mRNA expression of inflammatory markers, including *TNF*-α, *IL-1*β, and *IL-6*, and on the protein expression of p-IκB and p-NF-κB p65. Although the mRNA expression of *IL-1*β and *IL-6* was not significantly altered, the protein expression of p-IκB and p-p65 and the mRNA expression of *TNF*-α in the mammary glands of sheep were inhibited by rutin supplementation. These results indicate that rutin may inhibit the inflammatory response in the mammary glands of sheep during the transition period. This is consistent with the results of a previous study on mice (50).

In the bovine mammary gland, the sudden increase in metabolic activity, cellular proliferation, and physiological adaptations after calving all expose the mammary gland tissue to a considerable amount of ROS (31). To investigate the antioxidant effects of rutin on the mammary gland during the transition period in sheep, we quantified the expression of genes and proteins involved in the AMPK/NFE2L2 pathway. AMPK, a key protein kinase that regulates energy metabolism, is an upstream kinase of *NFE2L2* (51). *NFE2L2* is an important antioxidant protein that regulates many antioxidant genes such as *HO-1* and *NQO1* (52). Studies have shown that *NFE2L2* protects mammary epithelial cells against oxidative damage in ruminants *in vitro* (53). Our results showed that rutin significantly increased AMPK mRNA and protein expression. Although the total protein expression of *NFE2L2* was not affected by rutin, its mRNA expression was increased after 100 mg/kg rutin supplementation. Additionally, the levels of the downstream antioxidant genes *NFE2L2, NQ1*, and *HO-1* were also increased. These results suggest that rutin supplementation reduces oxidative damage in the mammary glands of sheep.

Additionally, we investigated the effects of rutin supplementation on the expression of apoptosis-related proteins in the mammary glands of sheep during the transition period. Caspase9 is an initiator caspase component of the apoptosome complex that plays a crucial role in activating effector caspases in response to a variety of death stimuli (54). Activated caspase9 cleaves and activates the downstream effector caspases such as caspase3, resulting in cell apoptosis. Furthermore, the intrinsic pathway includes the Bcl-2 family proteins (55). Bcl-2 is an anti-apoptotic protein that prevents apoptosis by suppressing mitochondrial membrane damage and blocking the release of cytochrome c (56). The activation of pro-apoptotic Bax translocates to the mitochondria, causing cytochrome c release and triggering apoptosis (55). The change in the Bax/Bcl-2 ratio seems to trigger the mitochondrial apoptotic pathway, resulting in caspase3 activation (57). In the present study, the protein expression of caspase9 and caspase3 and the ratio of Bax/Bcl-2 were significantly reduced after rutin supplementation in the mammary glands of sheep. Rutin supplementation significantly inhibited the expression of various apoptotic markers, which may improve the health status of the mammary glands of sheep.

## Conclusions

We found that rutin supplementation increased fat mobilization to provide energy and alleviate metabolic stress.

## Data Availability Statement

The original contributions presented in the study are included in the article/supplementary material, further inquiries can be directed to the corresponding author/s.

## Ethics Statement

The animal study was reviewed and approved by the Ethics Committee for Animal Care and Use of Anhui Agricultural University (Approval number 20210417).

## Author Contributions

HD and YL: analyzed the data and wrote the manuscript. YL, JW, and XW: project administration. JW and XW: designed the study and revised the manuscript. YL and CZ: revised the manuscript. YY, CX, DZ, and SF: participated in the acquisition of the data. HD, YY, CX, and DZ: performed the laboratory analysis. All authors contributed to the article and approved the submitted version.

## Funding

This work was supported by the National Natural Science Foundation of China (Beijing, China; Grant Nos. 32172924 and 31873029), Anhui Province Science Fund for Excellent Young Scholars, the Special Fund for Anhui Agriculture Research System (AHCYJSTX-07), Anhui Province Key Laboratory of Animal Nutrition Regulation and Health (APKLANRH202003), and Agricultural Industry Chief Expert Studio of Hefei City (dairy cows farming).

## Conflict of Interest

The authors declare that the research was conducted in the absence of any commercial or financial relationships that could be construed as a potential conflict of interest.

## Publisher's Note

All claims expressed in this article are solely those of the authors and do not necessarily represent those of their affiliated organizations, or those of the publisher, the editors and the reviewers. Any product that may be evaluated in this article, or claim that may be made by its manufacturer, is not guaranteed or endorsed by the publisher.
